# Grants Recommended by the Convalescent Homes Committee

**Published:** 1917-12-22

**Authors:** 


### Grants Recommended by the Convalescent Homes Committee.

### A.—CONSUMPTION SANATORIA.

*The Convalescent Homes Committee desire to draw attention to the fact that it must not be assumed that the reduction or absence of a grant implies dissatisfaction. (See paragraph 3 of Report.)*

**Children's Sanatorium, Holt, Norfolk,** £700, of which £650 in consideration of the reservation of ten children's beds during 1918 for the use of certain London hospitals (see paragraphs 5 and 6 of Report). **Daneswood Sanatorium (Jewish), Woburn Sands,** £400, of which £350 in consideration of the reservation of four beds during 1918 for the use of certain London hospitals (see paragraphs 5 and 6 of report). Devon and Cornwall Sanatorium, Didworthy, South Brent, £975, of which £875 in consideration of the reservation of ten beds during 1918 for the use of certain London hospitals (see paragraphs 5 and 6 of Report). **Eversfield Chest Hospital, St. Leonards,** £100, towards erection of cliff wall. **Fairlight Sanatorium, Hastings,** —. **Kelling Sanatorium, Holt, Norfolk,** £600, of which £525 in consideration of the reservation of six beds during 1918 for the use of certain London hospitals (see paragraphs 5 and 6 of Report). **Mount Vernon Hospital, Northwood,** £100. **National Association for the Establishment and Maintenance of Sanatoria for Workers, Benenden, Kent,** £2,975, of which £1,000 in reduction of mortgage debt, and £1,750 in consideration of the reservation of twenty beds during 1918 for the use of certain London hospitals (see paragraphs 5 and 6 of Report). Northamptonshire Sanatorium, Creaton, Northants, £1,200, of which £1,050 in consideration of the reservation of twelve beds during 1918 for the use of certain London hospitals (see paragraphs 5 and 6 of Report).

Total to Consumption Sanatoria, £7,050.

### B.—CONVALESCENT HOMES.

*The names of Convalescent Homes receiving no grant are not published.*

**Baldwin Brown Home for Convalescent Poor, Herne Bay,** £25. Beau Site Convalescent Home, Hastings, £25. Brentwood Convalescent Home for London Children, Brentwood, £50; Brooklands Homes (Invalid Children's Aid Association), Worthing, £25. Bushey and Bushey Heath Children's Convalescent Home Bushey, £25. **Chelsea Hospital for Women, St. Leonards,** £50, with a view to encouraging the admission without payment of patients direct from the hospital. Children's Cottage Hospital, Cold Ash, Newbury, £25, in consideration of convalescent cases. Children's Home Hospital, Barnet, £25, in consideration of convalescent cases. Convalescent Home for Poor Children, St. Leonards, £75. Convalescent Police Seaside Home, Hove, £50. **Friendly Societies' Convalescent Homes, Dover and Herne Bay,** £75. **Highgate Convalescent Home for Children, Highgate,** £25. **Jewish Convalescent Home (Children), Brighton,** £50. **King's College Hospital, Hemel Hempstead,** £75. **Metropolitan Convalescent Institution Bexhill,** £100. Metropolitan Convalescent Institution (Children), Broadstairs, £50, in consideration of work done during the early part of 1917. Metropolitan Convalescent Institution, Walton, £150. Miller Hospital Convalescent Home (late Deptford Medical Mission Convalescent Home), Bexhill, £25. **National Hospital for the Paralysed and Epileptic, East Finchley,** £100, with a view to facilitating the admission of patients without payment. New Hospital for Women (Home of Recovery), Barnet, £100. **Paddington Green Children's Hospital, Slough,** £125. Poplar Hospital, Walton-on-Naze, £50. **Queen Charlotte's Lying-in Hospital, Kilburn,** £50. Queen's Hospital for Children, Bexhill, £150. **Royal Waterloo Hospital for Children and Women, Whippingham,** £25. **St. Andrew's Convalescent Home, Folkestone,** £50. St. Andrew's Convalescent Hospital, Clewer, £25. St. Luke's Home (Children), Woodley, Reading, £25, in consideration of convalescent cases. St. Mary's Convalescent Home, Birchington, £25. St. Mary's Home for Children, Broadstairs, £75, in consideration of convalescent cases. St. Michael's Home for Men and Women, Westgate-on-Sea, £25. Sunday School Union Convalescent Home, Bournemouth, £25. Surgical Home for Boys, Banstead, £25, in consideration of convalescent cases. **Tilford Convalescent Home for Children, Farnham,** £25. **Victoria Home for Invalid Children, Margate,** £25, in consideration of convalescent cases. Victoria Hospital for Children, Chelsea, Broadstairs, £50, in consideration of work done during the early part of 1917. Victoria Hospital for Children, Chelsea, Biggin Hill, Kent, £25, in consideration of work done during the early part of 1917. Winifred House Invalid Children's Convalescent Hospital Home, Tollington Park, £25.

Total to Convalescent Homes, £1,950.

| | |
|---|---|
| Grants to Consumption Sanatoria ... | £7,050 |
| Grants to Convalescent Homes ... | ... £1,950 |
| Total for the Year 1917 ... | ... £9,000 |

